# Supplementary material for: Timing of iceberg scours and massive ice-rafting events in the subtropical North Atlantic
Source: Nat Commun. 2021 Jun 16;12:3668. doi: 10.1038/s41467-021-23924-0 (PMC8208987; doi:10.1038/s41467-021-23924-0)
Supplement: Supplementary file 1 — Supplementary Information [file 41467_2021_23924_MOESM1_ESM.pdf]

Supplementary Material

Timing of iceberg scours and massive ice-rafting events in the subtropical North Atlantic

Alan Condron[1*] and Jenna Hill[2]

[1]*Geology and Geophysics, Woods Hole Oceanographic Institution, Woods Hole, MA 02543, USA*

[2]*United States Geological Survey, Pacific Coastal & Marine Science Center Santa Cruz, CA 95005, USA*

*corresponding author: acondron@whoi.edu

Contents:

Supplementary Figure 1: Seafloor iceberg scours are observed as far south as the Florida Keys, with characteristic iceberg plough mark morphologies.
Supplementary Figure 2: The multi-level keel scheme used in the iceberg model to calculate ocean drag.
Supplementary Figure 3: The simulated distribution of icebergs in the glacial North Atlantic in response to a southward shift in the latitude of the Gulf Stream.
Supplementary Figure 4: Change in sea surface height in the subtropical western North Atlantic in response to elevated meltwater forcing from Hudson Bay, Canada.
Supplementary Figure 5: Simulated iceberg drift patterns in the western subtropical North Atlantic.
Supplementary Figure 6: Cross sections of salinity and meridional velocity at Florida Strait.
Supplementary Figure 7: Timeseries of the number of simulated icebergs in the North Atlantic.

Supplementary Table 1: Radiocarbon ages for all samples used in this study.
Supplementary Table 2: A list of the main coefficients used to derive iceberg motion.
Supplementary Table 3: A list of the main iceberg thermodynamics coefficients and constants.
Supplementary Table 4: Iceberg size distribution used in the model simulations.

Information on Supplementary Downloadable Data Files

Dataset1: Helpful files for plotting and reading binary data
XG.data: model longitude grid points (for plotting the binary output on a map projection)
YG.data: model latitude grid points (for plotting the binary output on a map projection)
readbin.m: Matlab script for reading all binary files. See comments below.

Dataset2: Data displayed in Figure 2
fig2_jan_SST.data: Sea surface temperature data (deg. C) for fig 2a.
fig2_sept_SST.data: Sea surface temperature data (deg. C) for fig 2b.
fig2_jan_uvel.data: u (zonal) component of surface ocean velocity (m/s) for fig. 2a
fig2_jan_vvel.data: v (meridional) component of surface velocity (m/s) for fig. 2a
fig2_sept_uvel.data: u (zonal) component of surface ocean velocity (m/s) for fig. 2b
fig2_sept_vvel.data: v (meridional) component of surface velocity (m/s) for fig. 2b

Dataset3: Data displayed in Figure 6
fig6_iceberg_density.txt: Iceberg density data displayed in Figure 6. 3 column table.

Dataset4: Data displayed in Figure 7
fig7_iceberg_locations.txt: Longitude/latitude locations of the icebergs displayed in Figure 7a-d.
fig7_SSS_PanelA.data: Sea surface salinity (SSS) data displayed in fig7a.
fig7_SSS_PanelB.data: Sea surface salinity (SSS) data displayed in fig7b.
fig7_SSS_PanelC.data: Sea surface salinity (SSS) data displayed in fig7c.
fig7_SSS_PanelD.data: Sea surface salinity (SSS) data displayed in fig7d.

Dataset5: Data displayed in Figure 8
fig8_SST_PanelA.data: Sea surface temperature (SST) data displayed in fig8a.
fig8_SST_PanelB.data: Sea surface temperature (SST) data displayed in fig8b.
fig8_SST_PanelC.data: Sea surface temperature (SST) data displayed in fig8c.
fig8d_SST_timeseries.txt: Time series of sea surface temperature shown in Fig 8d

Dataset6: Data displayed in Figure 9
fig9_iceberg_scour_data.txt: latitude and depth of the iceberg scours show in Fig 9

Notes: Data files ending '.data' are in binary format with 4-byte precision (real*4). All binary files (including the grid files) are two dimensional and are 3060x510 grid points in size. These files are all on the model's native grid with a spatial resolution of ~18km (1/6 deg.). The binary files can be read using the included MATLAB routine, readbin.m. For example, to read the sea surface

salinity data in file 'fig7_SSS_PanelA.data', in the Matlab command line type: sss=readbin('SSS.fig1_control.data', [3060 510]);

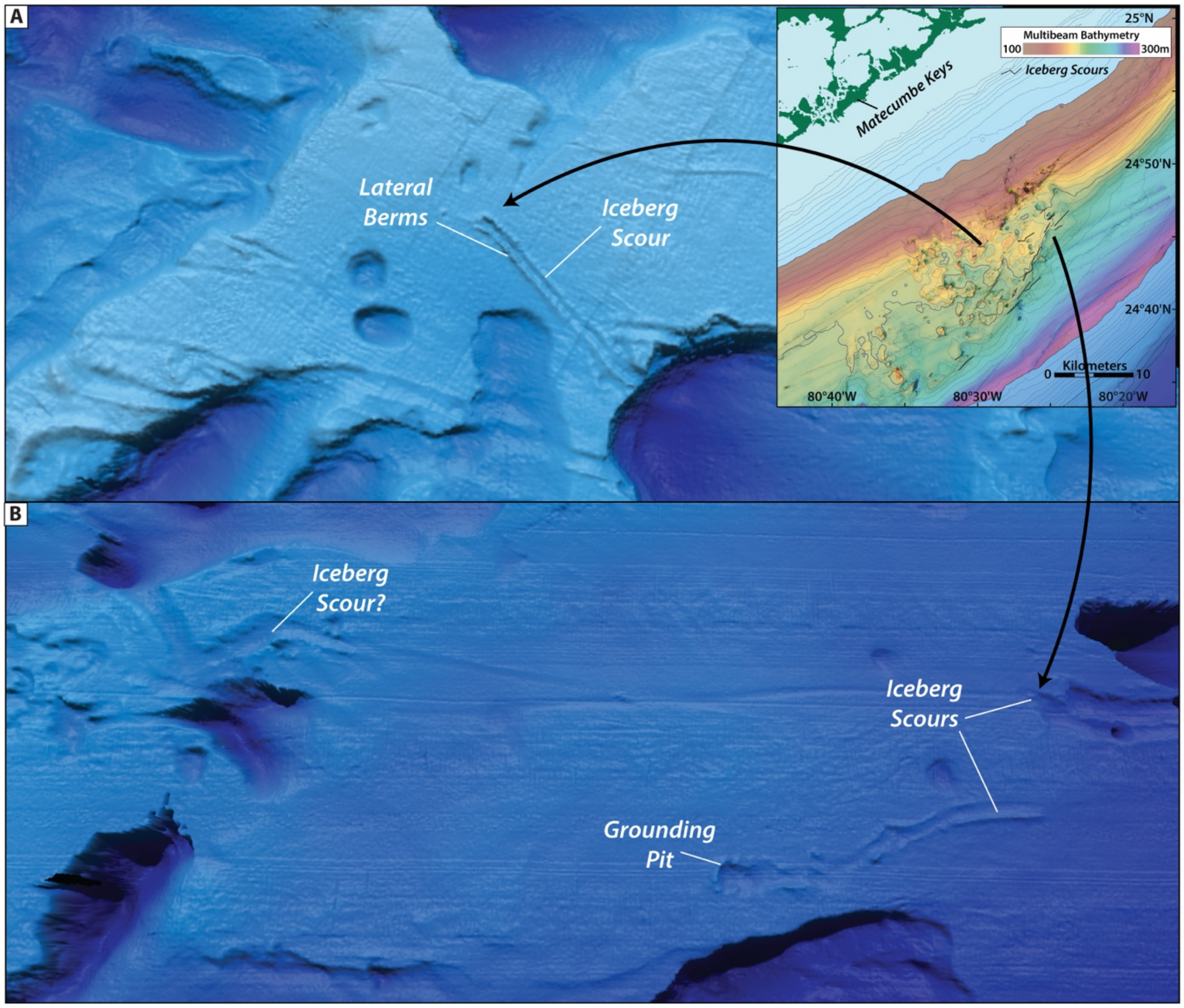

Supplementary Figure 1: Seafloor iceberg scours are observed as far south as the Florida Keys, with characteristic iceberg plough mark morphologies: (A) Lateral berms, interpreted as iceberg push-up ridges; (B) Terminal grounding pits indicate where icebergs came to rest on the seafloor

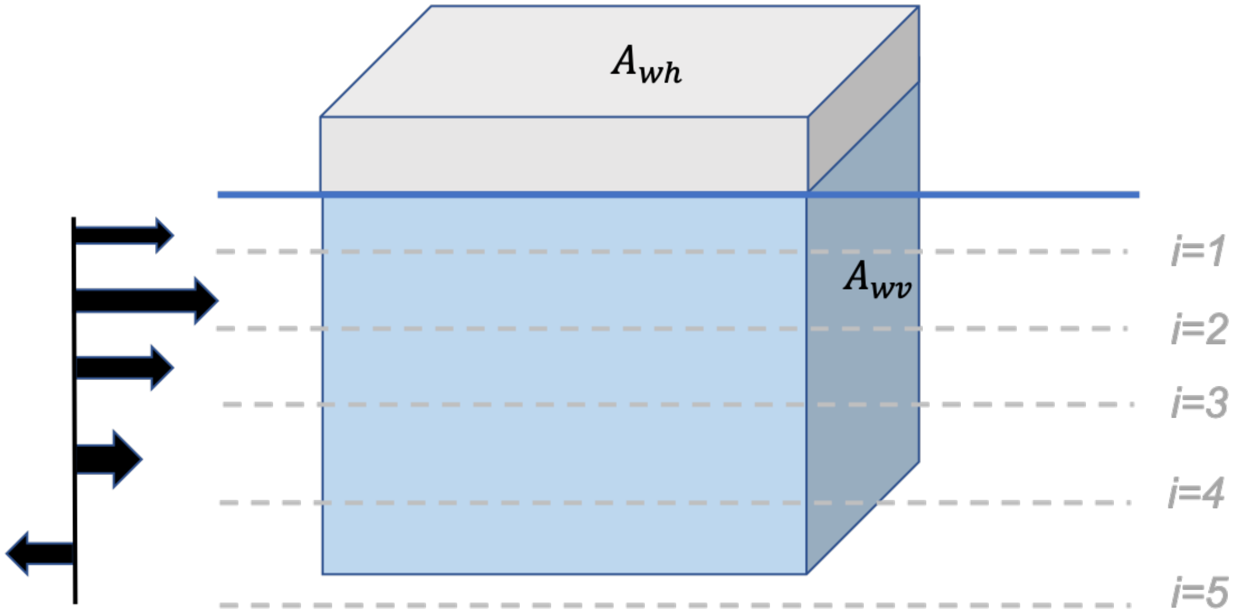

Supplementary Figure 2: The multi-level keel scheme used in the iceberg model to calculate ocean drag. In this example, the iceberg keel penetrates 5 vertical levels in the ocean model; flow in the top 4 levels is to the right of the page and to the left of the page in the bottom level. The net ocean drag exerted on the iceberg is the sum of all 5 levels.

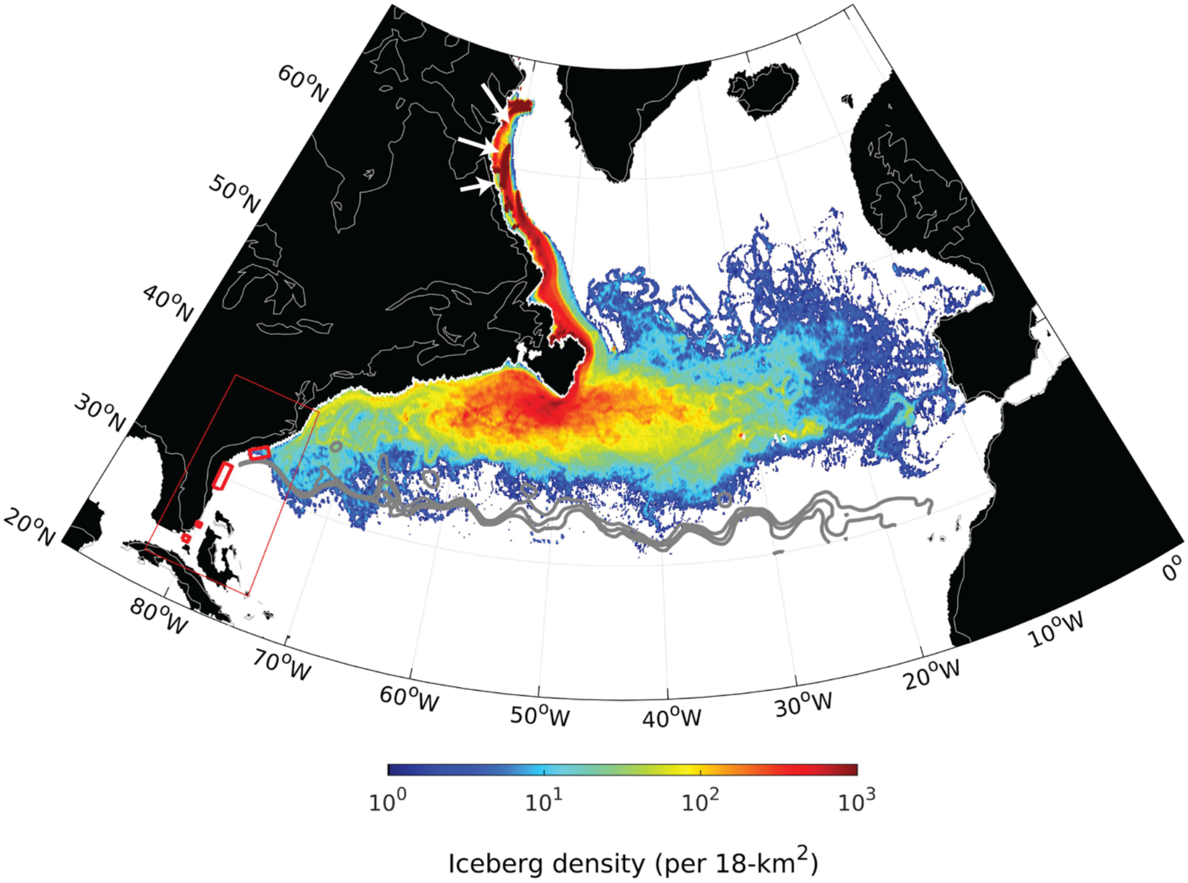

Supplementary Figure 3: The simulated distribution of icebergs in the glacial North Atlantic in response to a southward shift in the latitude of the Gulf Stream. Compared to the Control simulation (Fig. 5), a small number of icebergs drift to the most northern relic scour sites - located off the coast of South Carolina, USA - due to slope waters now flowing further south at Cape Hatteras. Icebergs were nevertheless still unable to reach the most southerly scour sites located off the coast of Florida that are directly beneath the northward flowing Gulf Stream. For reference, the Gulf Stream is marked by the 13-15°C isotherms at 200m water depth (grey contour lines). Iceberg calving margins near Hudson Bay are denoted by the white arrows, glacial landmasses are shown in black.

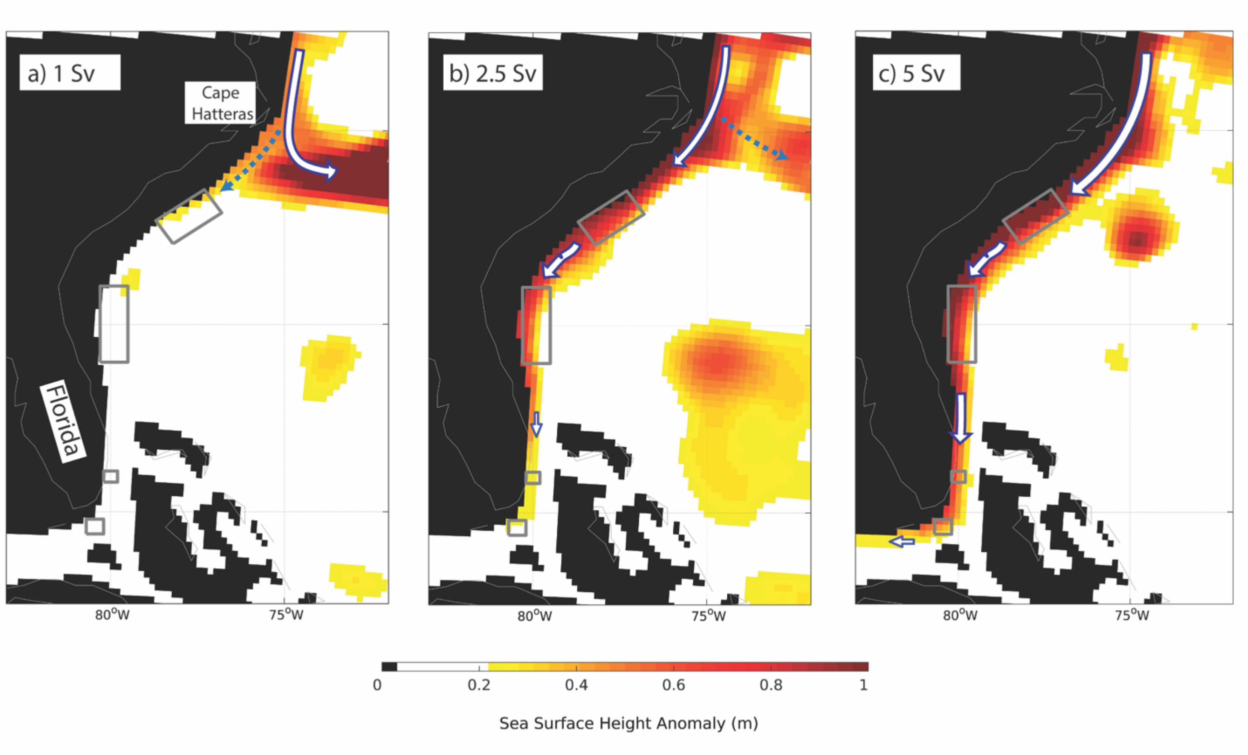

Supplementary Figure 4: Change in sea surface height in the subtropical western North Atlantic in response to elevated meltwater forcing from Hudson Bay, Canada. The panels (a-c) show the change in sea surface height (Perturbation minus Control) resulting from a 1 Sv, 2.5 Sv, and 5 Sv meltwater flood. The ability of the meltwater to flow south at Cape Hatteras, i.e. to 'overshoot', is dependant on whether the height of the meltwater exceeds the ambient sea surface height. This is the case for both the 2.5Sv and 5Sv meltwater floods, but not the 1Sv flood.

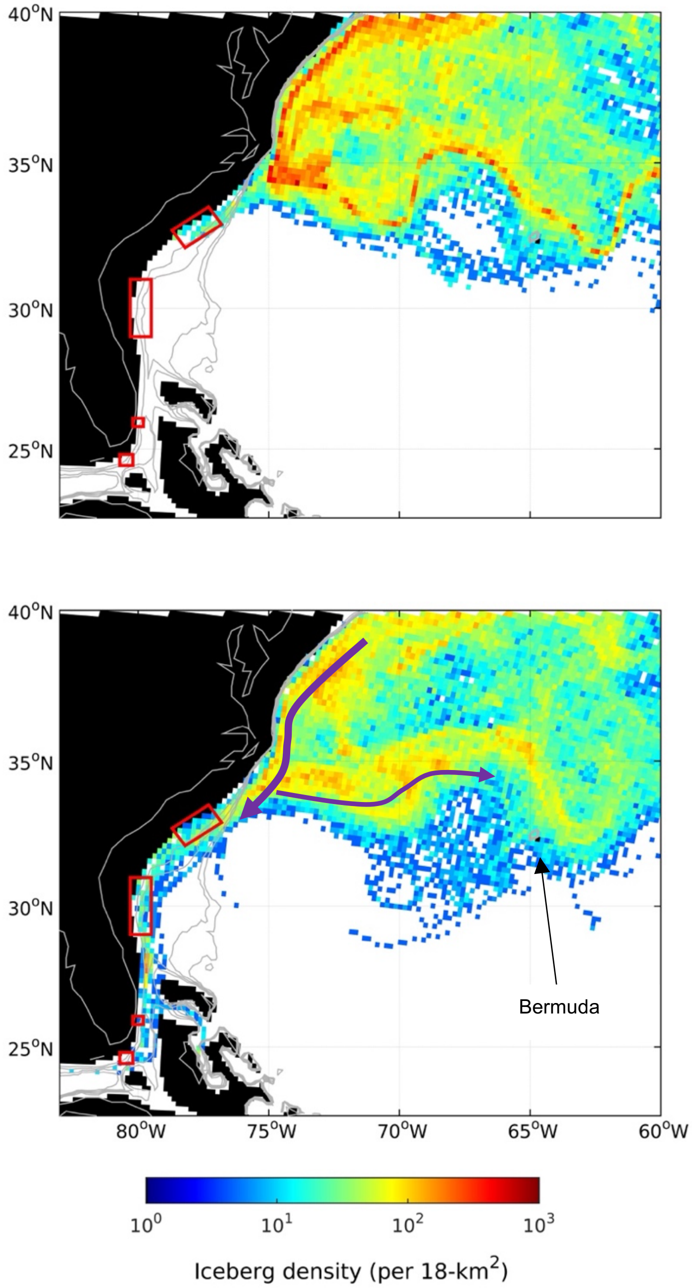

Supplementary Figure 5: Simulated iceberg drift patterns in the western subtropical North Atlantic. The maps show the mean density of icebergs for the first year of meltwater simulations with fluxes of 2.5 Sv (top) and 5 Sv (bottom). In the 2.5 Sv experiment, icebergs only reach the most northern relic subtropical scour sites off the coast of South Carolina; a flux of 5 Sv is required for icebergs to drift to the most southerly scours. The purple arrows (bottom panel) show the general drift directions of the icebergs: Initially, icebergs drift south along the eastern coast of the United States in the narrow coastal meltwater current; at Cape Hatteras a fraction of icebergs are

retroflected eastward into the interior of the subtropical Atlantic gyre, with a significant number reaching Bermuda. In the 5 Sv experiment, icebergs continue drifting along the east coast of the USA, as far south as Florida Keys.

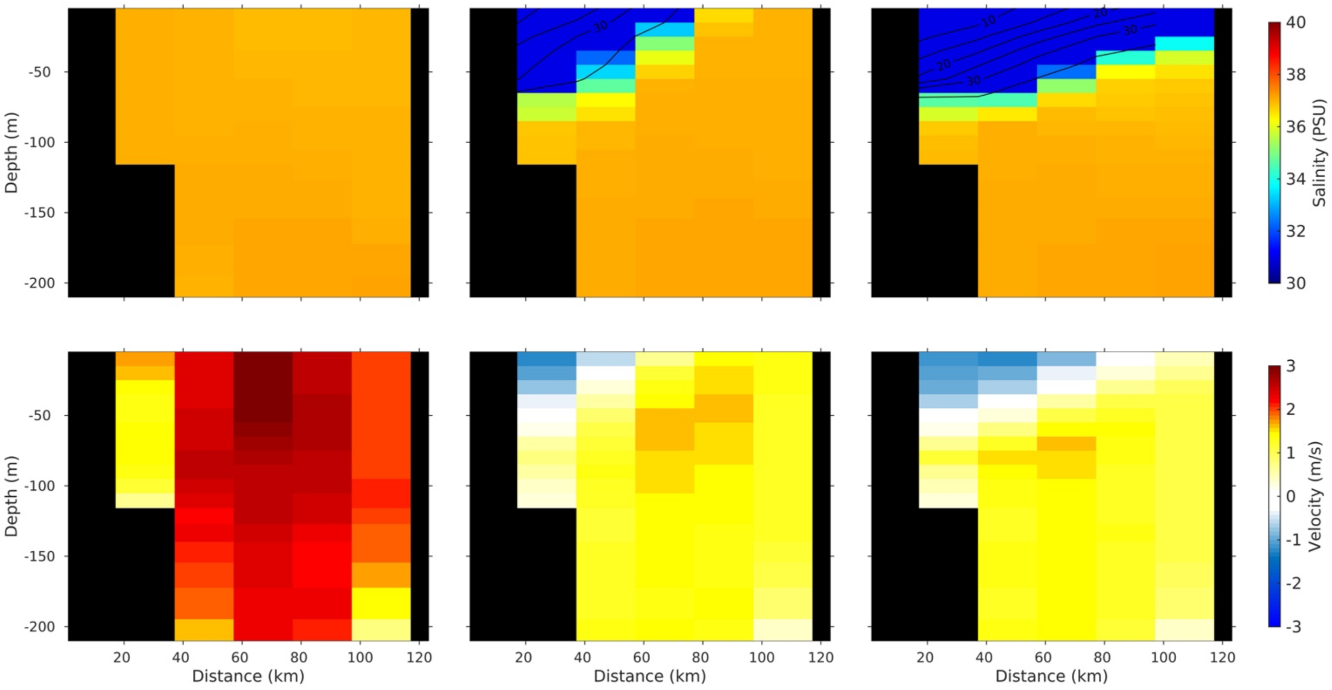

Supplementary Figure 6: Cross sections of salinity (top panels) and meridional (north-south) velocity (bottom panels) at Florida Strait (~26.5°N, 80°-78.5°W). The cross section is drawn as if the reader is looking north through the strait, such that the coast of Florida is on the left and Grand Bahama Island is to the right. The far-left panels show the salinity and flow in the Control, prior to the meltwater flood when flow is northwards at all depths. The two middle and two right panels show the ocean circulation in this region 90 and 300 days, respectively, after 5 Sv of meltwater was released from Hudson Bay.

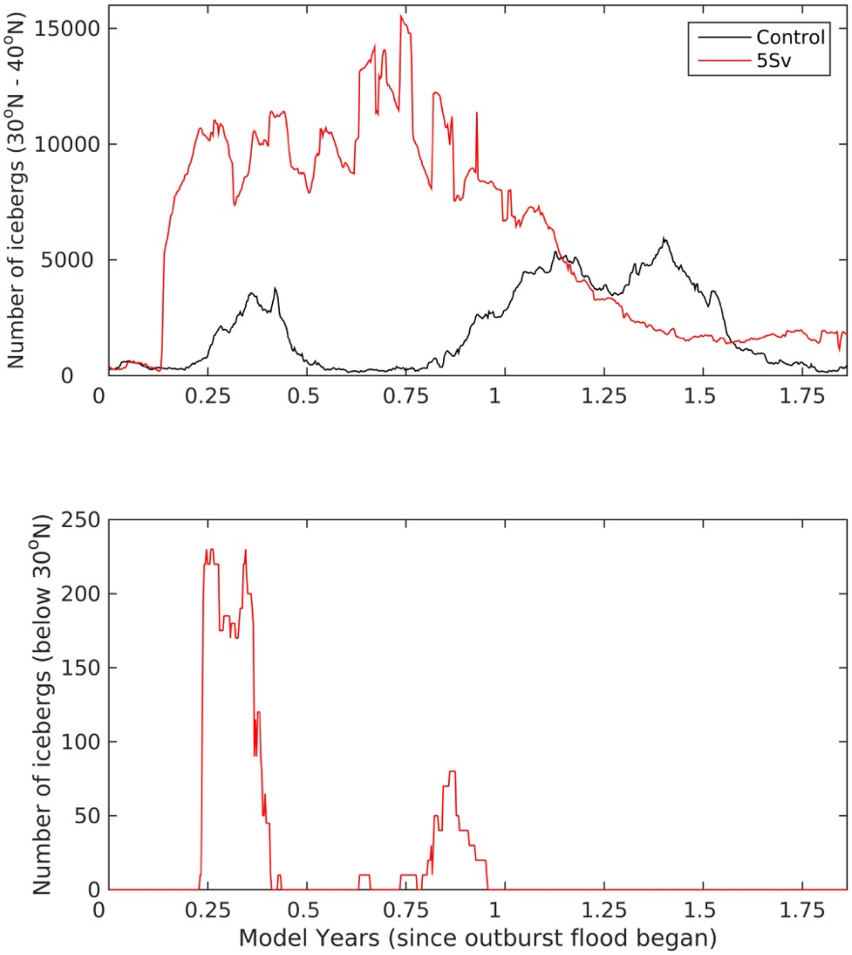

Supplementary Figure 7: Timeseries of the number of simulated icebergs in the North Atlantic between latitude bands 30° - 40°N (top) and below 30°N (bottom) in the Control and 5 Sv meltwater perturbation.

Supplementary Table 1: Radiocarbon ages for all samples used in this study.

Core	Sample Depth (cm)	Sample Type	^{14}C Age Yrs BP	Calendar Age Yrs BP	Heinrich Event*
24GC	68	lithology changes	11,830 ± 40	13,302	**YD**
24GC	116		15,300 ± 70	18,108	
24GC	125		27,150 ± 130	30,920	H3 ~31,000
24GC	129		26,800 ± 280	30,646	
24GC	150		17,920 ± 50	20,180	
24GC	152		17,130 ± 50	21,154	
20GC	106	above scour	24350 ± 90	27,990	
04GC	116	lithology change	22,100 ± 140	25,949	
04GC	120		22,800 ± 120	26,697	
04GC	124		24,500 ± 140	28131	
04GC	140	above scour	25,700 ± 250	29369	H3 ~31,000
04GC	142	below scour	28,500 ± 350	32014	
04GC	144		28,400 ± 350	31902	
02GC	115	lithology change	24,600 ± 150	28231	
02GC	116		25,900 ± 170	29612	
02GC	133	lithology change	24,000 ± 140	27717	
02GC	135		25,100 ± 160	28734	
02GC	142	above scour	26,500 ± 280	30393	H3 ~31,000
02GC	145	below scour	29,000 ± 250	32624	
02GC	160	above scour	28,500 ± 130	31846	H4 38,000
02GC	162	below scour	33,400 ± 230	37100	
02GC	165		42,300 ± 2800	45896	
27GC	130	above scour	28,200 ± 570	31863	H3 ~31,000
27GC	133	below scour	29,600 ± 680	33249	
27GC	208	lithology change	> 45000		
27GC	210		> 49400		
27GC	218	lithology change	> 45700		
27GC	220		> 45800		
27GC	225	lithology change	45,900 ± 2000		
27GC	226		51,500 ± 3900		
27GC	235	above scour	47,400 ± 1200		H4 38,000
27GC	236	below scour	35,000 ± 310	39118	
27GC	240		44,100 ± 830		
03GC	140	lithology change	> 52,000		
03GC	142		> 52,000		
03GC	166	above scour	51,200 ± 5900		H5 45,000
03GC	170	below scour	46,400 ± 3300		
03GC	174		47,400 ± 3700		

**nearest event in time - calendar ages from Hemming (2004)*

Supplementary Table 2: A list of the main coefficients used to derive iceberg motion.

Coefficient	Description	Units	Value
ρ_i	density of iceberg	kg/m^3	917
ρ_w	density of water	kg/m^3	1025
ρ_a	density of air	kg/m^3	1.2
ρ_s	density of sea ice	kg/m^3	910
C_{wv}	vertical drag coefficient for water	dimensionless	1
C_{av}	vertical drag coefficient for air	dimensionless	0.8
C_{sv}	vertical drag coefficient for sea ice	dimensionless	1
C_{wh}	horizontal drag coefficient for water	dimensionless	0.0012
C_{ah}	horizontal drag coefficient for air	dimensionless	0.0055
g	Gravity	m/s^2	9.8

Supplementary Table 3: A list of the main iceberg thermodynamics coefficients and constants.

Coefficient	Description	Units	Value
Γ_i	latent heat of fusion of ice	J/kg	3.33x10^5
T_i	Iceberg temperature	°C	-4
α	Iceberg albedo	dimensionless	0.7
k_a	Thermal conductivity of air (at 10°C)	J/s/m/K	0.0249
k_w	Thermal conductivity of water (at 0°C)	J/s/m/K	0.563
υ_a	kinematic viscosity of air (at 10°C)	m^2/s	1.46x10^{-5}
υ_w	kinematic viscosity of water (at 0°)	m^2/s	1.83x10^{-6}
D_a	thermal diffusivity air (at 0°C)	m^2/s	2.16x10^{-5}
D_w	thermal diffusivity water (at 0°C)	m^2/s	1.37x10^{-7}
R	Roughness height of the iceberg	m	0.01
W_p	Wave period	s	6.2

Supplementary Table 4: Iceberg size distribution used in the model simulations.

Size Class	Fraction (%)	Width (m)	Thickness (m)
1	15	67	80
2	15	133	160
3	20	200	240
4	15	267	320
5	8	333	360
6	7	400	360
7	5	500	360
8	5	600	360
9	5	800	360
10	5	1000	360
