# Supplementary material for: Timing of iceberg scours and massive ice-rafting events in the subtropical North Atlantic
Source: Nat Commun. 2021 Jun 16;12:3668. doi: 10.1038/s41467-021-23924-0 (PMC8208987; doi:10.1038/s41467-021-23924-0)
Supplement: Supplementary file 9 — Description of additional supplementary files [file 41467_2021_23924_MOESM9_ESM.docx]

Description of additional supplementary files

Title: Dataset1

Description: Helpful files for plotting and reading binary data XG.data: model longitude grid points (for plotting the binary output on a map projection) YG.data: model latitude grid points (for plotting the binary output on a map projection) readbin.m: Matlab script for reading all binary files. See comments below.

Title: Dataset2

Description: Data displayed in Figure 2 fig2_jan_SST.data: Sea surface temperature data (deg. C) for fig 2a. fig2_sept_SST.data: Sea surface temperature data (deg. C) for fig 2b. fig2_jan_uvel.data: u (zonal) component of surface ocean velocity (m/s) for fig. 2a fig2_jan_vvel.data: v (meridional) component of surface velocity (m/s) for fig. 2a fig2_sept_uvel.data: u (zonal) component of surface ocean velocity (m/s) for fig. 2b fig2_sept_vvel.data: v (meridional) component of surface velocity (m/s) for fig. 2b

Title: Dataset3

Description: Data displayed in Figure 6 fig6_iceberg_density.txt: Iceberg density data displayed in Figure 6. 3 column table.

Title: Dataset4

Description: Data displayed in Figure 7 fig7_iceberg_locations.txt: Longitude/latitude locations of the icebergs displayed in Figure 7a-d. fig7_SSS_PanelA.data: Sea surface salinity (SSS) data displayed in fig7a. fig7_SSS_PanelB.data: Sea surface salinity (SSS) data displayed in fig7b. fig7_SSS_PanelC.data: Sea surface salinity (SSS) data displayed in fig7c. fig7_SSS_PanelD.data: Sea surface salinity (SSS) data displayed in fig7d.

Title: Dataset5

Description: Data displayed in Figure 8 fig8_SST_PanelA.data: Sea surface temperature (SST) data displayed in fig8a. fig8_SST_PanelB.data: Sea surface temperature (SST) data displayed in fig8b. fig8_SST_PanelC.data: Sea surface temperature (SST) data displayed in fig8c. fig8d_SST_timeseries.txt: Time series of sea surface temperature shown in Fig 8d

Title: Dataset6

Description: Data displayed in Figure 9 fig9_iceberg_scour_data.txt: latitude and depth of the iceberg scours show in Fig 9 Notes: Data files ending ‘.data’ are in binary format with 4-byte precision (real*4). All binary files (including the grid files) are two dimensional and are 3060x510 grid points in size. These files are all on the model’s native grid with a spatial resolution of ~18km (1/6 deg.). The binary files can be read using the included MATLAB routine, readbin.m. For example, to read the sea surface 3 salinity data in file 'fig7_SSS_PanelA.data', in the Matlab command line type: sss=readbin('SSS.fig1_control.data', [3060 510]);
